# A Multiple-Trait Bayesian Variable Selection Regression Method for Integrating Phenotypic Causal Networks in Genome-Wide Association Studies

**DOI:** 10.1534/g3.120.401618

**Published:** 2020-10-05

**Authors:** Zigui Wang, Deborah Chapman, Gota Morota, Hao Cheng

**Affiliations:** * Department of Animal Science, University of California, Davis; † Department of Animal and Poultry Sciences, Virginia Polytechnic Institute and State University

**Keywords:** Structural Equation Models, Bayesian Regression, Variable Selection, GWAS, Genomic Prediction, GenPred, Shared data resources

## Abstract

Bayesian regression methods that incorporate different mixture priors for marker effects are used in multi-trait genomic prediction. These methods can also be extended to genome-wide association studies (GWAS). In multiple-trait GWAS, incorporating the underlying causal structures among traits is essential for comprehensively understanding the relationship between genotypes and traits of interest. Therefore, we develop a GWAS methodology, SEM-Bayesian alphabet, which, by applying the structural equation model (SEM), can be used to incorporate causal structures into multi-trait Bayesian regression methods. SEM-Bayesian alphabet provides a more comprehensive understanding of the genotype-phenotype mapping than multi-trait GWAS by performing GWAS based on indirect, direct and overall marker effects. The superior performance of SEM-Bayesian alphabet was demonstrated by comparing its GWAS results with other similar multi-trait GWAS methods on real and simulated data. The software tool JWAS offers open-source routines to perform these analyses.

Genome-wide association studies (GWAS) are widely used to identify associations between single nucleotide polymorphisms (SNPs) and phenotypes (Ozaki *et al.* 2002; Visscher *et al.* 2017; McCarthy *et al.* 2008; Cantor *et al.* 2010). GWAS have successfully mapped quantitative trait loci (QTL) associated with traits of interest, *e.g.*, meat quality and quantity in livestock (Sharma *et al.* 2015), crop yields in plants (Liu and Yan 2018), and diseases in humans (Visscher *et al.* 2017). GWAS are typically based on using linear mixed models to fit one SNP at a time to a single trait (Hackinger and Zeggini 2017). While this allows for a relatively simple statistical model, the interwoven nature of gene expression translates to many traits being correlated with each other (Sodini *et al.* 2018). These correlations can be utilized in multi-trait linear mixed models for GWAS to reduce false positives and increase the statistical power for association mapping (O’Reilly *et al.* 2012; Korte *et al.* 2012).

Conventional multi-trait linear mixed models do not consider the causal relationships between traits. To address this issue, researchers have proposed refining the multi-trait methods with structural equation models (SEM) introduced by Wright (1934) that consider the causal relationship among traits. A model that incorporates causal structures should better reflect underlying genetic mechanisms. Gianola and Sorensen (2004) used SEM to extend conventional multi-trait linear mixed models to accommodate for recursive and simultaneous relationships among traits, which allows traits to be explanatory variables for other traits. Recently, Momen *et al.* (2018, 2019) proposed the SEM-based GWAS (SEM-GWAS) methodology by applying SEM to linear mixed models for GWAS. They showed that while conventional GWAS methodology only provides overall SNP effects, SEM-GWAS can capture the complex causal relationships among traits and further decompose the overall SNP effects into direct and indirect effects.

The SEM-GWAS method proposed by Momen *et al.* (2018, 2019) is based on linear mixed models with a fixed substitution effect for the tested SNP and a random effect with covariances defined by a “genomic relationship matrix” computed from genotypes (VanRaden 2008) to account for genetic relatedness. Markers are usually implicitly assumed to affect all traits when the ”genomic relationship matrix” is constructed in multi-trait analysis. However, this assumption is not biologically meaningful, especially in multi-trait analyses involving many traits. Cheng *et al.* (2018b) proposed a general class of multi-trait Bayesian variable selection regression methods that use a broad range of mixture priors, *e.g.*, multi-trait BayesCΠ, where each locus can affect any combination of traits, which allows us to more closely model the true biological mechanisms, *e.g.*, pleiotropy (Cheng *et al.* 2018b).

The primary goal of this current research is to develop a multi-trait Bayesian regression GWAS method that more closely resembles the underlying biological mechanisms including pleiotropy and causal structure among traits. In this paper, we develop and implement a new GWAS method called SEM-Bayesian alphabet, which integrates SEM to the multi-trait Bayesian variable selection methods, to incorporate the underlying biological mechanism. The term “Bayesian alphabet” denotes a collection of Bayesian regression models that differ in the priors adopted for marker effects (Gianola 2013). In this paper, we use SEM-BayesCΠ, a Bayesian variable selection method, to show the utility of the SEM-Bayesian alphabet. The performance of our proposed method is studied using real and simulated data.

## Materials And Methods

### Multi-trait Bayesian regression model using mixture priors

Assuming that individuals have all traits measured with a general mean as the only fixed effect, we write the multi-trait model for individual *i* from *n* genotyped individuals as:

$$\begin{array}{cc} y_{i} & =\mu +\overset{p}{\underset{j=1}{\sum }}m_{ij}\alpha_{j}+e_{i} \end{array}$$

where $y_{i}$ is the vector of phenotypes of *t* traits for individual *i*, μ is a vector of overall means for *t* traits, *p* is the number of genotyped loci, $m_{ij}$ is the genotype covariate at locus *j* for individual *i* (coded as 0,1,2), $\alpha_{j}$ is the vector of marker effects of *t* traits for locus *j*, and $e_{i}$ is the vector of residuals of *t* traits for individual *i*. The fixed effects are assigned flat priors. The residuals, $e_{i}$, are a priori assumed to be independently and identically distributed multivariate normal vectors with null mean and covariance matrix R, which is assumed to have an inverse Wishart prior distribution, $W^{-1}\left(S_{e},\nu_{e}\right)$.

Allowing each locus to affect any combination of traits, in a multiple-trait Bayesian variable selection method, *e.g.*, multi-trait BayesCΠ (Cheng *et al.* 2018b), the vector of marker effects at locus *j* can be written as $\alpha_{j}=D_{j}\beta_{j}$, where $D_{j}$ is a diagonal matrix whose diagonal element is $\delta_{j}=\left(\delta_{j1},\delta_{j2}\ldots ,\delta_{jt}\right)$, where $\delta_{jk}$ is the indicator variable indicating whether the marker effect of locus *j* for trait *k* is zero or not, and $\beta_{j}$ is a priori assumed to be independently and identically distributed multivariate normal vectors with null mean and covariance matrix G, which is assumed to have an inverse Wishart prior distribution, $W_{t}^{-1}\left(S_{\beta },\nu_{\beta }\right)$. Given that a locus can have an effect on any combination of traits, we use numeric labels ″1″,″2″,⋯,″l″ to represent all $2^{t}$ possible combinations for $\delta_{j}$, in which case the prior distribution for $\delta_{j}$ is:

$$p\left(\delta_{j}=\prime \prime i\prime \prime \right)={\text{Π}}_{1}I\left(\delta_{j}=\prime \prime 1\prime \prime \right)+{\text{Π}}_{2}I\left(\delta_{j}=\prime \prime 2\prime \prime \right)+\ldots +{\text{Π}}_{l}I\left(\delta_{j}=\prime \prime l\prime \prime \right)$$

where ${\text{Π}}_{i}$ is the prior probability that the vector $\delta_{j}$ corresponds to the vector labeled “*i*” and $\sum^{\text{​}}{\text{Π}}_{i}=1$. We assume the prior for $\text{Π}=\left({\text{Π}}_{1},{\text{Π}}_{2},\ldots {\text{Π}}_{l}\right)$ is a uniform distribution.

### Structural Equation Model

The linear SEM is composed of two parts: the measurement equation analyzing the relationship between the observable variables and latent variables, and the structural equation capturing the connections among latent variables (Anderson and Gerbing 1988). These two equations can be written as:

$$\left\{\begin{array}{cc} y_{i} & =\Lambda \left(\begin{array}{c} \eta_{i}\\ \xi_{i} \end{array}\right)+\kappa_{1}\;\text{measurement equation}\\ \eta_{i} & =\Gamma_{1}\eta_{i}+\Gamma_{2}\xi_{i}+\kappa_{2}\;\text{structural equation} \end{array}\right.$$

where $y_{i}$ is the vector of observable variables for individual *i*, $\eta_{i}$ is a q×1 vector of endogenous latent variables, $\xi_{i}$ is a r×1 vector with exogenous latent variables, $\Gamma_{1}$ and $\Gamma_{2}$ are the matrix of unknown coefficients in structural equation, Λ is a $t\times \left(q+r\right)$ matrix of unknown structural coefficients, $\kappa_{1}$ and $\kappa_{2}$ are t×1 and *q* × 1 vectors of residuals. The details of parameter estimation are discussed in Song and Lee (2012).

In our study, no latent variables are assumed and the sole observable variables are phenotypes. Thus only the causal relationship among observable variables, *i.e.*, phenotypes, are fitted in the SEM model (also known as path analysis (Wright 1921)) as:

$$\begin{array}{cc} y_{i} & =\Lambda y_{i}+\varepsilon_{i} \end{array}$$ (1)

where $y_{i}$ and Λ are defined as above, $\varepsilon_{i}$ represents everything that is not explained by $\Lambda y_{i}$, and Λ is an t×t matrix of structural coefficients representing the causal structure recovered from the Inductive Causation (IC) algorithm as described in the next section.

To illustrate, we assume that the phenotypes of three traits for each individual (*i.e.*, $y_{1},y_{2},$ and $y_{3}$ for traits 1, 2, and 3) have the following causal relationship:

$$\left\{\begin{array}{cc} y_{1}=\varepsilon_{1} & \\ y_{2}=\lambda_{12}y_{1}+\varepsilon_{2} & \\ y_{3}=\lambda_{13}y_{1}+\lambda_{23}y_{2}+\varepsilon_{3} & \end{array}\right.$$

where causal coefficient $\lambda_{ij}$ represents that a 1-unit increase in trait *i* results in a $\lambda_{ij}$ unit increase in trait *j*. Given the causal structure above, the Λ can be written as:

$$\Lambda =\left(\begin{array}{ccc} 0 & 0 & 0\\ \lambda_{12} & 0 & 0\\ \lambda_{13} & \lambda_{23} & 0 \end{array}\right)$$ (2)

### Searching causal structure

As described above, fitting the SEM requires the causal structure among all traits to be known before analysis. To explore the wide-range of possible underlying causal structures, we use the method from Valente *et al.* (2010) to discern the causal structure based on the posterior distribution of the residual covariance matrix. The reason we do not directly apply this method to phenotype data are that the covariance among phenotypes is likely confounded by genetic effects. The process of inferring causal structure is composed of three steps:

1. Fit the multi-trait BayesianCΠ model and obtain the posterior distribution of the residual covariance matrix.
2. Follow Valente *et al.* (2010) to derive the conditional independence relationship among traits based on the posterior distribution of the residual covariance matrix. In detail, we derive the residual partial correlation p($y_{i}$, $y_{j}$|h), where h is a set of traits, to test whether trait $y_{i}$ is conditionally independent from $y_{j}$. The highest posterior density (HPD) interval of 0.9 was used to make statistical decisions. If HPD interval of p($y_{i}$, $y_{j}$|h) contains zero, $y_{i}$ and $y_{j}$ are regarded as conditionally independent on h.
3. Apply the IC algorithm (Pearl 2009) as described in the Appendix to the conditional independence relationship from step 2 to obtain the causal structure.

### SEM-BayesCΠ

Assume $\varepsilon_{i}=\mu +{\sum^{\text{​}}}_{j=1}^{p}m_{ij}\alpha_{j}+e_{i}$ in equation (1) and follow assumptions in multi-trait BayesCΠ, the SEM-BayesCΠ model can be written as:

$$\begin{array}{cc} y_{i} & =\Lambda y_{i}+\mu +\overset{p}{\underset{j=1}{\sum }}m_{ij}D_{j}\beta_{j}+e_{i} \end{array}$$ (3)

Move $\Lambda y_{i}$ from the right side to the left side of equation (3), and define $\Lambda^{\text{*}}=I-\Lambda$, where I is a t×t identity matrix and Λ is a t×t matrix of structural coefficients based on the discerned causal structure, the model becomes:

$$\begin{array}{cc} \Lambda^{\text{*}}y_{i} & =\mu +\overset{p}{\underset{j=1}{\sum }}m_{ij}D_{j}\beta_{j}+e_{i} \end{array}$$ (4)

To guarantee that the structural coefficient is identifiable, we assume that the residuals for each trait of individual *i* are independent with each other, which means the residual covariance matrix is diagonal (Wu *et al.* 2010; Momen *et al.* 2018). The vector of all non-zero elements in Λ, *e.g.*, $\lambda =\left[\lambda_{12},\lambda_{13},\lambda_{23}\right]$, is assumed to have a prior distribution:

$$\lambda |\lambda_{0},\tau^{2}∼N\left(1\lambda_{0},I\tau^{2}\right)$$

where **1** is a vector of ones, I is the identity matrix, and $\lambda_{0}$ is a known mean for all elements in λ. $\tau^{2}$ is a tuning parameter to adjust the sharpness degree of the prior (Gianola and Sorensen 2004). In this paper, we set $\lambda_{0}=0$ and $\tau^{2}=1$. The priors for the remaining parameters are the same as in the section Multi-trait Bayesian regression model using mixture priors.

Gibbs samplers are used to draw samples for all parameters. The full conditional distribution to draw samples for λ is shown below. The derivations of the full conditional distributions of the remaining parameters of interest for Gibbs samplers are in Cheng *et al.* (2018b).

*Full conditional distribution of* Λ***:*** We follow Gianola and Sorensen (2004) to obtain the full conditional distribution of Λ, with the difference between our derivation and Gianola and Sorensen (2004) being that we specify the causal structure with positions of parameters in the Λ. Let Ω denote all parameters except λ in the SEM-BayesCΠ and use the causal structure $\Lambda =\left(\begin{array}{ccc} 0 & 0 & 0\\ \lambda_{12} & 0 & 0\\ \lambda_{13} & \lambda_{23} & 0 \end{array}\right)$ as an example, the left hand side of equation (4), $\Lambda^{\text{*}}y_{i}$, can be written as:

$$\begin{array}{l} \Lambda^{*}y_{i}=\left(\begin{array}{ccc} 1 & 0 & 0\\ -\lambda_{12} & 1 & 0\\ -\lambda_{13} & -\lambda_{23} & 1 \end{array}\right)\left(\begin{array}{c} y_{i1}\\ y_{i2}\\ y_{i3} \end{array}\right)\\ =\left(\begin{array}{c} y_{i1}\\ y_{i2}-\lambda_{12}y_{i1}\\ y_{i3}-\lambda_{13}y_{i1}-\lambda_{23}y_{i2} \end{array}\right)\\ =\left(\begin{array}{c} y_{i1}\\ y_{i2}\\ y_{i3} \end{array}\right)\text{-}\left(\begin{array}{ccc} 0 & 0 & 0\\ y_{i1} & 0 & 0\\ 0 & y_{i1} & y_{i2} \end{array}\right)\left(\begin{array}{c} \lambda_{12}\\ \lambda_{13}\\ \lambda_{23} \end{array}\right)\\ =y_{i}-Y_{i}\lambda \end{array}$$

The conditional posterior distribution of λ can be written as:

$$\begin{array}{l} p(\lambda |\Omega ,y)\propto \prod_{i=1}^{n}N(y_{i}|\Lambda^{*-1}\left(\mu +\sum_{j=1}^{p}m_{ij}D_{j}\beta_{j}),\Lambda^{*-1}R\Lambda^{*-1\prime }\right)\\ N(\lambda |1\lambda_{0},I\tau^{2})\\ \propto |\Lambda^{*}{|}^{\frac{n}{2}}\prod_{i=1}^{n}N(\Lambda^{*}y_{i}|\mu +\sum_{j=1}^{p}m_{ij}D_{j}\beta_{j},R)N\left(\lambda |1\lambda_{0},I\tau^{2}\right)\\ =|\Lambda^{*}{|}^{\frac{n}{2}}exp[-\frac{1}{2}\sum_{i=1}^{n}(\Lambda^{*}y_{i}-\mu -\sum_{j=1}^{p}m_{ij}D_{j}\beta_{j}{)}^{\prime }R^{-1}\\ \left(\Lambda^{*}y_{i}-\mu -\sum_{j=1}^{p}m_{ij}D_{j}\beta_{j}\right)]\\ \times exp\left[-\frac{1}{2\tau^{2}}{\left(\lambda -1\lambda_{0}\right)}^{\prime }\left(\lambda -1\lambda_{0}\right)\right] \end{array}$$ (5)

Setting $w_{i}=y_{i}-\mu -{\sum^{\text{​}}}_{j=1}^{p}m_{ij}D_{j}\beta_{j}$, equation (5) can be written as:

$$\begin{array}{l} p(\lambda |\Omega ,y)∼|\Lambda^{*}{|}^{\frac{n}{2}}exp\left[-\frac{1}{2}\sum_{i=1}^{n}(w_{i}-Y_{i}\lambda {)}^{\prime }R^{-1}(w_{i}-Y_{i}\lambda )\right]\\ {}*exp\left[-\frac{1}{2\tau^{2}}{\left(\lambda -1\lambda_{0}\right)}^{\prime }\left(\lambda -1\lambda_{0}\right)\right] \end{array}$$

Following the derivation in Gianola and Sorensen (2004) and the fact that $\left|\Lambda^{*}\right|=1$ in a recursive system, the full conditional distribution of λ is

$$p(\lambda |\Omega ,y)∼N\left(\hat{\lambda },V_{\lambda }\right),$$

where

$$\begin{array}{cc} \hat{\lambda } & ={\left(\overset{n}{\underset{i=1}{\sum }}Y_{i}\prime R^{-1}Y_{i}+\tau^{-2}I\right)}^{-1}\left(\overset{N}{\underset{i=1}{\sum }}Y_{i}\prime R^{-1}w_{i}+\tau^{-2}1\lambda_{0}\right)\\ V_{\lambda } & ={\left(\overset{n}{\underset{i=1}{\sum }}Y_{i}\prime R^{-1}Y_{i}+\tau^{-2}I\right)}^{-1} \end{array}$$

### Decomposition of SNP effects

In SEM-BayesCΠ, the marker effect for locus *j*, $\alpha_{j}$, is considered as the vector of direct marker effect of *t* traits. The indirect effect of locus *j* of *t* traits can be calculated as ${\sum^{\text{​}}}_{\rho =1}^{t-1}\Lambda^{\rho }\alpha_{j}$. The overall effect of locus *j* on *t* traits is computed as ${\sum^{\text{​}}}_{\rho =0}^{t-1}\Lambda^{\rho }\alpha_{j}$ or ${\left(I-\Lambda \right)}^{-1}\alpha_{j}$, which is the summation of both direct and indirect effect of locus *j*. For example, given a causal structure $\Lambda =\left(\begin{array}{ccc} 0 & 0 & 0\\ \lambda_{12} & 0 & 0\\ \lambda_{13} & \lambda_{23} & 0 \end{array}\right)$, the direct effect for locus *j* on three traits is $\alpha_{j}=\left(\begin{array}{c} \alpha_{1j}\\ \alpha_{2j}\\ \alpha_{3j} \end{array}\right)$, and the indirect effect for locus *j* on three traits is calculated as

$$\Lambda \alpha_{j}+\Lambda^{2}\alpha_{j}=\left(\begin{array}{c} 0\\ \lambda_{12}\alpha_{1j}\\ \left(\lambda_{13}+\lambda_{12}\lambda_{23}\right)\alpha_{1j}+\lambda_{23}\alpha_{2j}. \end{array}\right),$$

and the overall effect of locus *j* on trait *k* is $\left(\begin{array}{c} \alpha_{1j}\\ \lambda_{12}\alpha_{1j}+\alpha_{2j}\\ \left(\lambda_{13}+\lambda_{12}\lambda_{23}\right)\alpha_{1j}+\lambda_{23}\alpha_{2j}+\alpha_{3j} \end{array}\right)$.

### Inference of association based on genomic windows

Markers in a genomic window are usually highly correlated, indicating that any single marker may not show a strong association with the trait even though a causal variant exists in the window. In this paper, we make an inference of association based on genomic windows, because multiple markers inside a genomic window may jointly capture the signal from the causal variant (Fernando and Garrick 2013; Fernando *et al.* 2017).

To make an inference of association based on genomic windows, posterior distribution for the proportion of the genetic variance explained by markers in genomic window *w*, $q_{w}$, is estimated from MCMC samples of overall, direct, and indirect marker effects as follows. For one MCMC sample of all marker effects on one trait, let $\alpha_{direct}$, $\alpha_{indirect}$, and $\alpha_{overall}$ denote direct, indirect, and overall effects of all markers respectively.

The genetic value that is attributed to genomic window *w* is calculated as:

$$\begin{array}{cc} a_{w,direct} & =M_{w}\alpha_{w,direct}\\ a_{w,indirect} & =M_{w}\alpha_{w,indirect}\\ a_{w,overall} & =M_{w}\alpha_{w,overall} \end{array}$$

where $M_{w}$ is a matrix of marker covariates in window *w* and $\alpha_{w,direct}$, $\alpha_{w,indirect}$, and $\alpha_{w,overall}$ are the MCMC samples of direct, indirect, and overall marker effects for SNPs in window *w*. Then the variance explained by the genomic window *w* is defined as:

$$\begin{array}{l} \sigma_{a_{w,direct}}^{2}=\frac{a_{w,direct}^{T}a_{w,direct}}{n}-(\frac{1_{n}^{T}a_{w,direct}}{n}{)}^{2}\\ \sigma_{a_{w,indirect}}^{2}=\frac{a_{w,indirect}^{T}a_{w,indirect}}{n}-(\frac{1_{n}^{T}a_{w,indirect}}{n}{)}^{2}\\ \sigma_{a_{w,overall}}^{2}=\frac{a_{w,overall}^{T}a_{w,overall}}{n}-(\frac{1_{n}^{T}a_{w,overall}}{n}{)}^{2} \end{array}$$

Similarly, the total genetic variance is calculated as:

$$\begin{array}{cc} \sigma_{a}^{2} & =\frac{a_{overall}^{T}a_{overall}}{n}-{\left(\frac{1_{n}^{T}a_{overall}}{n}\right)}^{2} \end{array}$$

The proportion of the genetic variance explained by direct, indirect, and overall marker effects in the genomic window *w* is calculated as:

$$\begin{array}{cc} q_{w,direct} & =\frac{\sigma_{a_{w,direct}}^{2}}{\sigma_{a}^{2}}\\ q_{w,indirect} & =\frac{\sigma_{a_{w,indirect}}^{2}}{\sigma_{a}^{2}}\\ q_{w,overall} & =\frac{\sigma_{a_{w,overall}}^{2}}{\sigma_{a}^{2}} \end{array}$$

Given the MCMC samples of $q_{w}$, the window posterior probability of association (WPPA) is calculated as the proportion of MCMC samples of $q_{w}$ that exceed a specific value *T* (Fernando and Garrick 2013; Chen *et al.* 2017; Lloyd-Jones *et al.* 2017). In this paper, associations are tested for non-overlapping windows of 100 SNPs, and genomic windows that explain over $\frac{1}{N}$ of the total genetic variance were deemed to be of potential interest (*i.e.*, $T=\frac{1}{N}$, where *N* is the total number of windows).

### Data analysis

#### Real data:

The Rice Diversity Panel with 413 *Oryza sativa* individual accessions was used in the analysis. Three traits were considered, including plant height (PH), flowering time in Arkansas (FTA), and panicle number per plant (PN) in our GWAS. After removing the records with missing data for these three traits and genotype with minor allele frequency <0.05, 370 individuals with 33,519 SNPs genotyped were included in our analysis. The phenotypic and genotypic data were publicly available for download from http://www.ricediversity.org/. It has been shown that using a threshold of WPPA =α to declare a significant genomic window restricts the proportion of false positives (PFP) to <1−α (Fernando *et al.* 2017). A previous GWAS (Zhao *et al.* 2011) identified significantly associated SNPs in chromosome 6 for flowering time in Arkansas (FTA) using the same dataset. A threshold of WPPA = 0.8 and p-value = 5$\times 10^{-6}$ in our GWAS analysis resulted in similarly significant signals. This result suggests that a WPPA of 0.8 and p-value = 5$\times 10^{-6}$ are reasonable for declaring a significant genomic window.

#### Simulated data:

**Figure 1:**
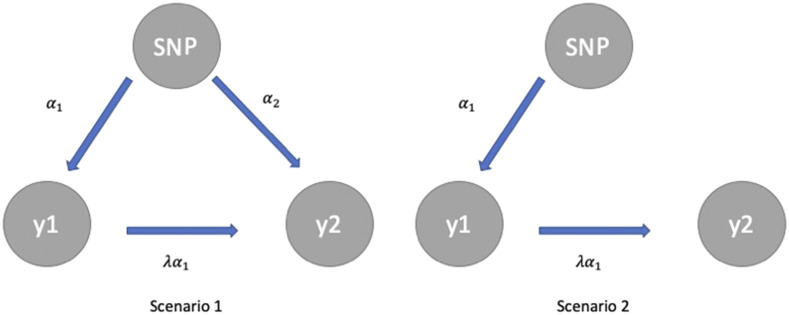
$\alpha_{1}$: the direct effect on trait $y_{1}$; $\alpha_{2}$: the direct effect on trait $y_{2}$; $\lambda \alpha_{1}$: the indirect effect on trait $y_{2}$. The graph shows the simulated two QTL effect simulation scenarios. Scenario 1 represents that the QTL has direct effect on both trait 1 and trait 2, whereas scenario 2 represents that the QTL only have direct effect on trait 1.

To compare SEM-BayesCΠ with SEM-GWAS of Momen *et al.* (2018), we simulated data based on real genotypes from the Rice Diversity Panel. The simulation scenarios in Chen *et al.* (2017) were applied to simulate different genetic architectures. The QTL effects were generated from unit-gamma distribution (scale = 1) with three different shape parameters (*γ*): fewer QTL with large effects (γ=0.18), fewer QTL with small or large effects and many QTL with intermediate effects (γ=3.0), and the intermediate case (γ=1.48). In addition to the distribution of QTL effects, the number of QTL ($n_{QTL}$) may play an important role in GWAS, thus three numbers of QTL ($n_{QTL}=30,90,300$) combined with the three shape parameters were used to create 9 scenarios. For each scenario, 50 replicated populations were simulated with QTL positions randomly sampled across the genome. Trait 1 was assumed to have a causal effect on trait 2 with causal structural coefficient λ=1.0. The QTL effects were simulated under two scenarios (Figure 1): the QTL have direct effect on both trait 1 and trait 2 in scenario 1, where QTL only have direct effect on trait 1 in scenario 2. Half of the QTL were simulated following scenario 1, while the remaining followed scenario 2. Phenotypes for two traits were generated based on heritability of 0.5. In the simulated data analysis, the causal structure was assumed known to exclude the bias caused by searching causal structures. The structural coefficients were assumed known in SEM-GWAS.

We have implemented SEM-Bayesian alphabet in JWAS (Cheng *et al.* 2018a), an open-source, publicly available package for single-trait and multi-trait whole-genome analyses written in the freely available Julia language. More details can be found at https://reworkhow.github.io/JWAS.jl/latest/.

## Results

### Simulated data result

The performances of SEM-BayesCΠ and SEM-GWAS were compared based on the AUC (Area under Receiver Operating Characteristic). Inference of association on genomic windows for SEM-GWAS was based on the minimum p-value (Begum *et al.* 2016), *i.e.*, genomic windows containing at least one significant variant are declared as significant windows. To exclude the irrelevant AUC with low levels of specificity, only the partial area under the curve up until the false positive rate of 5% (pAUC5) (Chen *et al.* 2017; Ma *et al.* 2013) was calculated. For the convenience of comparison, all pAUC5 measurements were rescaled such that the pAUC5 of the random classifier is equal to 1. The R package ROCR (Sing *et al.* 2005) was used to obtain the pAUC5; the paired *t*-tests (p-value < 0.1) were used for comparing both the pAUC5 mean across all scenarios (overall mean comparison) and the pAUC5 mean for each level of $n_{QTL}$ and shape parameters *γ* (marginal mean comparison).

The GWAS results based on overall, direct and indirect effect were shown in Table 1. For the overall effect result on trait 1, there is no significant difference between SEM-BayesCΠ and SEM-GWAS in both overall mean comparison and marginal mean comparison. The direct effect on trait 1 is the same as the overall effect on trait 1 since the direct effect and overall effect on trait 1 are equal based on the causal structure. For the overall effect on trait 2, the pAUC5 mean of SEM-BayesCΠ is significantly higher than that of SEM-GWAS in the overall mean comparison, and some marginal mean comparisons (*e.g.*, $n_{QTL}=30$ and γ=0.18). For the direct effect on trait 2, though higher overall mean of pAUC5 is usually observed in SEM-BayesCΠ, there is no significant difference (p-value < 0.1) between SEM-BayesCΠ and SEM-GWAS in both overall mean comparison and marginal mean comparison. For the indirect effect result on trait 2, similar to the overall effect result on trait 2, the pAUC5 mean of SEM-BayesCΠ is significantly higher than that of SEM-GWAS in the overall mean comparison, and some marginal mean comparisons (*e.g.*, $n_{QTL}=30$ and γ=0.18).

**Table 1:** Overall and marginal mean of rescaled pAUC5 of SEM-BayesCΠ and SEM-GWAS based on overall,direct and indirect effect

		Overall effect		Direct effect		Indirect effect	
	factors	SEMBayesCП	SEM-GWAS	SEMBayesCП	SEM-GWAS	SEMBayesCП	SEM-GWAS
trait 1	*n_QTL_*						
	30	4.99	4.66	4.99	4.66	NA	NA
	90	2.00	1.91	2.00	1.91	NA	NA
	300	1.38	1.42	1.38	1.42	NA	NA
	shape (γ)						
	0.18	2.71	2.52	2.71	2.52	NA	NA
	1.48	2.93	2.79	2.93	2.79	NA	NA
	3.00	2.72	2.69	2.72	2.69	NA	NA
	overall	2.79	2.66	2.79	2.66	NA	NA
trait 2	*n_QTL_*						
	30	5.49^‡^	4.74^†^	4.22	3.91	4.88^‡^	3.15^†^
	90	2.09	2.08	1.87	1.87	1.91	1.72
	300	1.34	1.38	1.30	1.33	1.28	1.30
	shape (γ)						
	0.18	3.10^‡^	2.80^†^	2.48	2.36	2.79^‡^	2.29^†^
	1.48	3.03	2.84	2.56	2.38	2.63	2.46
	3.00	2.84	2.60	2.36	2.38	2.64	2.43
	overall	2.99^‡^	2.74^†^	2.46	2.37	2.69^‡^	2.39^†^

For both trait 1 and trait 2, comparisons between methods are made for different number of QTL (*n_QTL_*) and shape parameters of QTL effects (γ). Estimations are based on 450 simulated data sets including nine scenarios discussed in Simulated data. For each effect, in each row, the values with different symbols have significantly different (p-value <0.1) pAUC5. NA represented pAUC5 was not available because the indirect effect on trait 1 does not exist based on the causal structure. The overall effect result on trait 1 is the same as the direct effect result on trait 1 because the overall effect on trait 1 equals the direct effect on trait 1 based on the causal structure.

### Real data result

#### Causal structure and structural coefficients:

**Figure 2:**
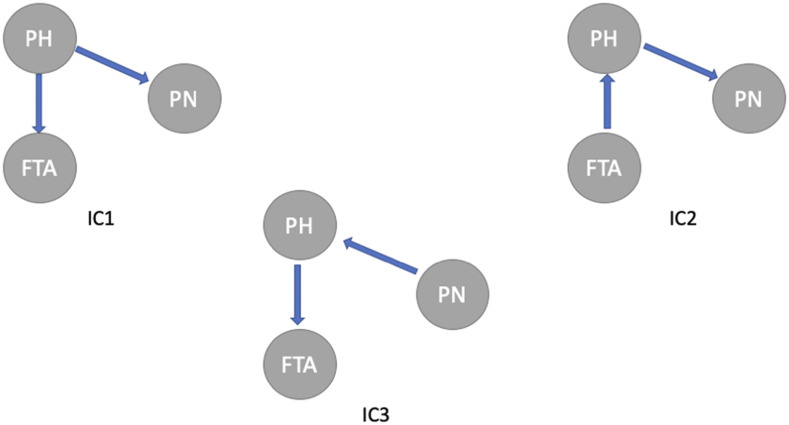
Causal structures among plant height (PH), flowering time in Arkansas (FTA), and panicle number per plant (PN) inferred from the IC algorithms. The edges connecting two traits represent non-null partial correlations as indicated by 0.9 HPD interval. The arrows represent the direction of causal effects.

The causal structure among three traits is inferred by the IC algorithm from the estimated posterior distribution of the residual covariance matrix in the multi-trait BayesCΠ model. Figure 2 shows three potential phenotypic causal structures among traits PH ($y_{1}$), FTA ($y_{2}$), and PN ($y_{3}$) recovered for the 0.9 HPD interval. The causal structure matrices for IC1 ($\Lambda_{1}$), IC2 ($\Lambda_{2}$), and IC3 ($\Lambda_{3}$) are:

$$\Lambda_{1}=\left(\begin{array}{ccc} 0 & 0 & 0\\ \lambda_{12} & 0 & 0\\ \lambda_{13} & 0 & 0 \end{array}\right),\Lambda_{2}=\left(\begin{array}{ccc} 0 & \lambda_{21} & 0\\ 0 & 0 & 0\\ \lambda_{13} & 0 & 0 \end{array}\right),\Lambda_{3}=\left(\begin{array}{ccc} 0 & 0 & \lambda_{31}\\ \lambda_{12} & 0 & 0\\ 0 & 0 & 0 \end{array}\right).$$

These three causal structures are fitted in the SEM-BayesCΠ model, and samples from posterior distributions for coefficients in these causal structures are obtained. The 90% credible intervals for structural coefficients in IC1, IC2, and IC3 are shown in Table 2. It is worth noting that the causal structures in Figure 2 provides the same set of marginal and conditional independencies, extra biological knowledge is required to further infer the causal structure. In this paper, the SEM-BayesCΠ model is proposed to incorporate (known) underlying causal structure among traits for GWAS. Thus, for the simplicity of our presentation, the causal structure is assumed known in Real Data Result section, *e.g.*, IC1 is used to demonstrate the performance of SEM-BayesCΠ.

**Table 2:** The 90% credible interval for causal structural coefficients in the three causal structures

λ	IC1	IC2	IC3
λ_PH→FTA_	(0.30, 0.52)	NA	(0.30, 0.55)
λ_PH→PN_	(-0.21, -0.02)	(-0.20, -0.01)	NA
λ_FTA→PH_	NA	(0.22, 0.44)	NA
λ_PN→PH_	NA	NA	(-0.27, -0.05)

PH, FTA, and PN represent traits plant height, flowering time in Arkansas, panicle number per plant, respectively. λ*_a_*_→_*_b_* represents the causal effects of trait *a* on trait *b*. NA denotes structural coefficients those do not exist in the causal structure.

#### Decomposition of SNP effects:

Direct, indirect, and overall SNP effects for all markers are estimated from SEM-BayesCΠ and SEM-GWAS. In SEM-BayesCΠ, direct SNP effects are assigned mixture priors, where each locus can affect any combinations of traits directly; samples from posterior distributions of indirect effects are obtained using joint samples from posterior distributions of Λ and direct SNP effects $\alpha_{j}$. In IC1, for trait PH, the overall SNP effect is equal to the direct SNP effect, because there is no intermediate trait. For trait FTA, the overall SNP effect is composed of direct SNP effect and indirect SNP effect transmitted from PH. So the overall SNP effect for FTA is given by summing the direct SNP effect and indirect SNP effect. Similarly, for trait PN, the overall SNP effect is obtained by summing the direct SNP effect and indirect SNP effect transmitted from PH.

**Figure 3:**
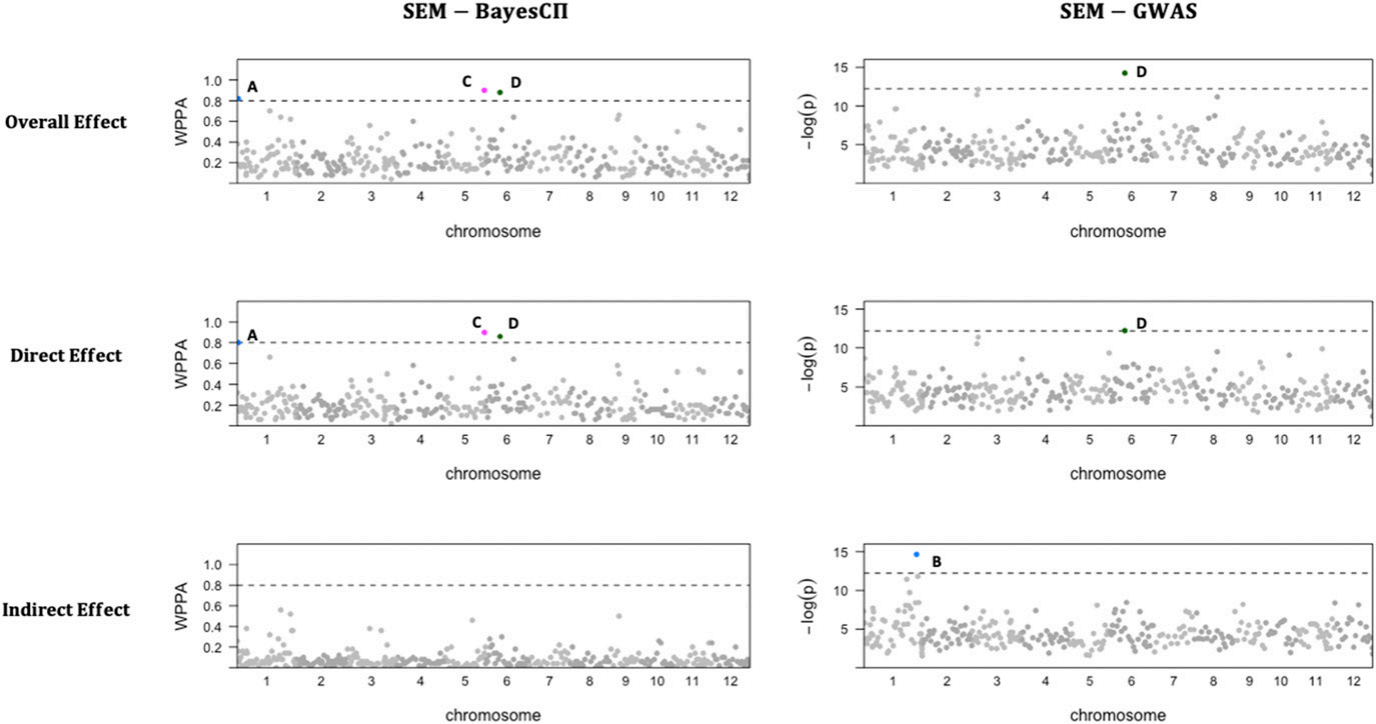
GWAS results based on overall, direct and indirect SNP effects from SEM-BayesCΠ and SEM-GWAS incorporating IC1 causal structure for the trait flowering time at Arkansas (FTA). The horizontal dash line represents the threshold 0.8 or -log($5\times 10^{-6}$). X-axis represents the location of genomic windows along the 12 chromosomes; Y-axis represents window posterior probability of association (WPPA) for SEM-BayesCΠ and negative logarithm of the p-value (-log(p)) for SEM-GWAS. Colored points represent genomic windows with WPPA ≥ 0.8 or p-value $\leq 5\times 10^{-6}$. The blue points, pink points and green points represents the significant genomic windows located in chromosome 1, chromosome 5 and chromosome 6.

The results of GWAS from SEM-BayesCΠ and SEM-GWAS incorporating causal structure IC1 are shown in Figure 3. Significant signals are found only for trait FTA. SEM-BayesCΠ adopts a threshold of WPPA = 0.8 to declare a significant genomic window and SEM-GWAS adopts a threshold of p-value = $5\times 10^{-6}$. The overall SNP effects are partitioned into direct and indirect effects, and GWAS are performed for the direct, indirect, and overall SNP effects separately for trait FTA. In Figure 3, the blue points, pink points and green points represents the significant genomic windows located in chromosome 1, chromosome 5 and chromosome 6. Window A contains SNPs from “id1000759” to “id1001229”; window B contains SNPs from “id1023967” to “id1024499”; window C contains SNPs from “id5013234” to “id5013920”; window D contains SNPs from “id6005814” to “id6006470”.

For the overall effects, in the SEM-GWAS, window D achieved -log(p-value) 14.21; in the SEM-BayesCΠ, window C achieved WPPA 0.90, window D achieved WPPA 0.88, and window A achieved WPPA 0.82. For the direct effect, in the SEM-GWAS, window D achieved -log(p-value) 12.24; in the SEM-BayesCΠ, window C achieved WPPA 0.90, window D achieved WPPA 0.86, and window A achieved WPPA 0.80. For the indirect effect, in the SEM-GWAS, window B achieved -log(p-value) 14.65; in the SEM-BayesCΠ, although no window is identified as significant in SEM-BayesCΠ, a peak was observed at window B with WPPA 0.52. Further, for all three effects, the results from SEM-BayesCΠ and SEM-GWAS are correlated (the correlation between the WPPA from SEM-BayesCΠ and -log(p-value) from the SEM-GWAS is higher than 0.5). The correlation of indirect effect results from these two methods results achieved 0.70. Also, for both SEM-BayesCΠ and SEM-GWAS, the overall effect is more correlated with direct effect rather than indirect effect. The magnitudes for overall, direct, and indirect SNP effect in SEM-BayesCΠ are also shown in Figure 5. Though most large overall SNP effects consist of a large direct SNP effect and a relatively small indirect SNP effect, the indirect effect of some SNPs play an important role, *e.g.*, the overall effect of SNP “id1024159”, as shown in Figure 5, consists of a large indirect SNP effect and relatively small direct effect.

### Genetic pleiotropy in SEM-BayesCΠ

**Figure 4:**
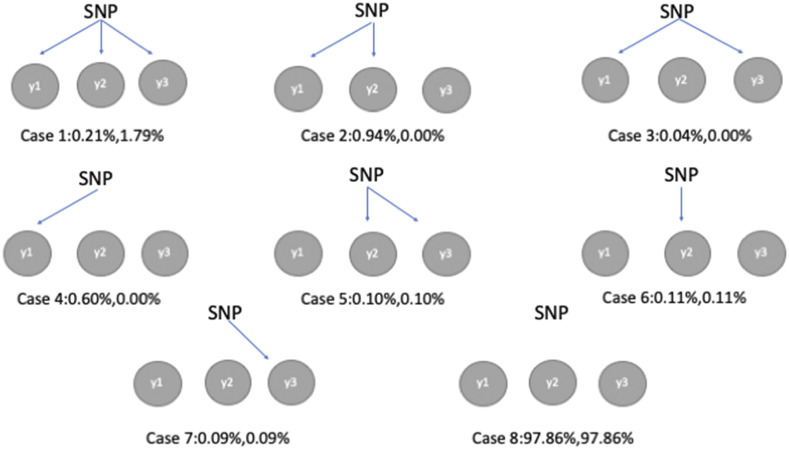
Estimated proportion of markers affecting combinations of traits, Π, from SEM-BayesCΠ incorporating IC1 causal structure. For each scenario, it is estimated for both direct SNP effects (left) and overall SNP effects (right).

As shown in Figure 4, the posterior distribution of the parameter Π is obtained, and markers show different levels of pleiotropy for direct SNP effects. In SEM-BayesCΠ, each SNP can have direct effects on any combination of traits, and the parameter Π is used to estimate the proportion of SNPs having different levels of pleiotropy. Indirect SNP effects on one trait are transmitted from direct SNP effects on other intermediate traits. For example, in causal structure IC1, the indirect SNP effects on FTA is transmitted from direct SNP effects on the trait PH. A SNP having no direct effect on FTA may have overall effects on FTA if its direct effect on trait PH is non-zero. The proportions of markers affecting different combinations of traits through overall SNP effects can also be estimated. This result is shown in Figure 4, and different probabilities are observed for some cases between overall and direct SNP effects. More SNPs have effects on all traits simultaneously when overall SNP effects are considered compared to direct SNP effects (case 1). If only overall SNP effects are considered, some cases having non-zero probabilities for indirect SNP effects are hidden by the causal relationships among traits (cases 2-4). The same patterns for direct and overall SNP effects are observed in cases 5-8 because there is no causal relationship between trait FTA and PN.

## Discussion

The complex causal relationships among multiple traits are usually not considered in conventional multi-trait GWAS. Here we propose the SEM-Bayesian alphabet method to incorporate pre-inferred causal structures among multiple traits into multi-trait Bayesian regression methods. SEM-Bayesian alphabet accounts for causal structures among traits, and has the potential advantage of estimating causal effects, providing genomic window-based inference, as well as providing a comprehensive understanding of the underlying biological mechanism.

### GWAS

To show the potential utility of SEM-Bayesian alphabet, simulated data were used to compare SEM-BayesCΠ with SEM-GWAS. A wide variety of potential genomic architectures were constructed by the combination of different levels of skewness of gamma distribution for QTL effects (*γ*) and different numbers of QTL($n_{QTL}$) (Chen *et al.* 2017).

**Figure 5:**
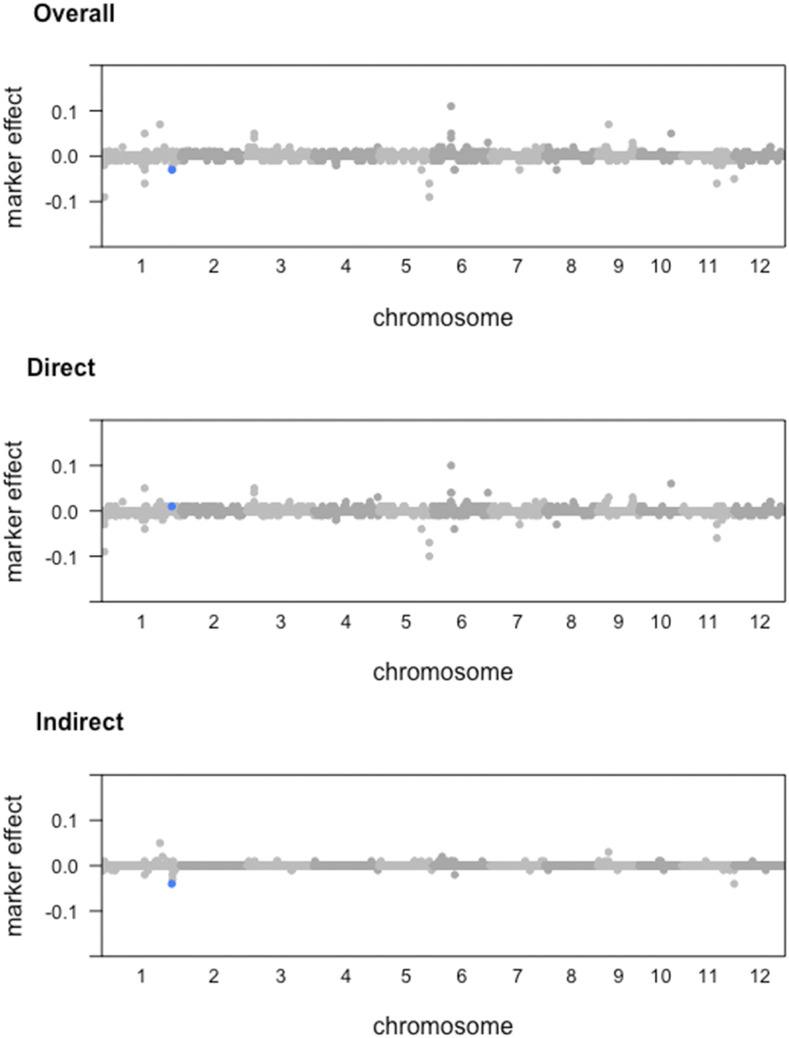
Magnitude of direct, indirect and overall SNP effect from SEM-BayesCΠ incorporating IC1 causal structure for the trait flowering time at Arkansas (FTA). X-axis represents the location of SNPs along the 12 chromosomes. Y-axis represents the magnitude of the marker effects. The blue points represents the SNP ”id1024159”. For SNP “id1024159”, the overall effect is consists of a small direct SNP effect and a relatively large indirect SNP effect.

BayesCΠ was also performed to estimate overall SNP effects on the same datasets, and similar results as those in the SEM-BayesCΠ were obtained (not shown in this paper). This is reasonable since the SEM-Bayesian alphabet model can be reduced to a model similar to Bayesian regression by reparameterization, indicating that the joint likelihood functions of SEM-Bayesian alphabet and Bayesian regression are similar. Compared to Bayesian regression, the SEM-Bayesian alphabet provides a more comprehensive understanding of the underlying biological mechanism by decomposing overall SNP effects into direct and indirect SNP effects (Figures 3, 4 and 5).

The comparison between SEM-BayesCΠ and SEM-GWAS using simulated data were shown in Table 1. As shown in our results, SEM-BayesCΠ has relatively the same or higher pAUC5 than SEM-GWAS in all simulation scenarios. In some scenarios, SEM-BayesCΠ has significantly higher pAUC5 than SEM-GWAS. For example, when one trait is affected by few QTL of large effects (*e.g.*, *n_QTL_* = 30,$shape\left(\gamma =0.18\right)$), SEM-BayesCΠ has significantly higher pAUC5 than SEM-GWAS to infer indirect and overall effects. Though significant difference is not observed for direct effect, higher overall mean of pAUC5 is usually observed in SEM-BayesCΠ.

### Causal structure

The causal structure is assumed to be known in SEM-Bayesian alphabet, and it is usually discerned by three types of algorithms: the constraint-based algorithm, the score-based algorithms, and the hybrid algorithms. The IC algorithm (Pearl 2009; Valente *et al.* 2010) used in this paper is a typical constraint-based algorithm, which is based on conditional independence tests. The score-based algorithms apply the heuristic optimization techniques, which set an initial graph structure and assign an initial goodness-of-fit score to it, and then maximize the goodness-of-fit score to obtain the most possible causal structure. The hybrid algorithm is a hybrid of both the constraint-based and the score-based algorithms. It utilizes conditional independence tests to reduce the space of candidate causal structures, and uses network scores to identify the optimal structure among them (Scutari 2014). The causal structures inferred from these algorithms may be different. Note that different evaluation criteria may also result in different outcome causal structures. For example, in this paper, if we choose 0.99 instead of 0.9 HPD interval to search for causal structures, there will be no edge between the traits PH and PN.

### Decomposition of SNP effects

In some previous analysis (Mi *et al.* 2010; Momen *et al.* 2018, 2019), the indirect SNP effect of locus *j* of *t* traits is obtained by multiplying the estimated Λ, $\hat{\Lambda }$, and estimated direct SNP effects, $\hat{\alpha_{j}}$, as ${\sum^{\text{​}}}_{\rho =1}^{t-1}{\hat{\Lambda }}^{\rho }\hat{\alpha_{j}}$. This is similar to using posterior means of causal structural coefficients and direct SNP effects for calculation of the indirect SNP effects. In our method, indirect SNP effects are estimated using joint samples from posterior distributions of Λ and $\alpha_{j}$. We compared these two approaches for indirect SNP effect estimation on real rice data, and found that the indirect effects estimated from these two approaches are slightly different. The SEM-BayesCΠ approach should be used in indirect SNP effect estimation due to the fact that Λ and $\alpha_{j}$ may be highly dependent.

## Conclusion

SEM-Bayesian alphabet provides more interpretation into biological mechanisms than Bayesian regression methods by decomposing the overall SNP effects into direct and indirect SNP effects. In SEM-Bayesian alphabet, posterior distributions of the overall, direct, and indirect SNP effects, as well as causal structure coefficients, are obtained, which are used to make inferences about these parameters. Compared to the typical GWAS method incorporating causal structure among multiple traits, such as SEM-GWAS, SEM-Bayesian alphabet obtains the posterior distributions for the proportion of variance attributed to a genomic region to detect causal loci (*i.e.*, the use of WPPA). The level of gene pleiotropy, *e.g.*, proportion of markers affecting different combinations of traits as shown in Figure 4, can also be further dissected into direct and indirect SNP effects. Also, with estimating structural coefficients, SEM-Bayesian alphabet still has relatively same or greater pAUC5 than SEM-GWAS in all scenarios of simulated data. In summary, SEM-Bayesian alphabet offers a more comprehensive understanding of the underlying biological mechanisms including pleiotropy and causal relationships among traits than conventional GWAS, as well as has a potential advantage in the GWAS inference than other GWAS considering complex causal effect among multiple traits.

## APPENDIX

### Inductive causation algorithm

We use the IC algorithm (Pearl 2009) to recover the causal structure among a set of traits, denoted as *U* below, from the conditional independence relationship. The IC algorithm is composed of three steps (Valente *et al.* 2010; Chicharro and Panzeri 2014):

1. For each pair of traits *X* and *Y*, search for a set $S_{XY}\subseteq U$ such that $X⊥Y|S_{XY}$ holds. That is, X and Y are independent, conditional on $S_{XY}$. If there is no such $S_{XY}$, place an undirected edge between these two traits.

2. If this pair of traits *X* and *Y* are non-adjacent (*i.e.*, no un-directed edge between *X* and *Y*) with a common neighbor *Z* (*i.e.*, Z is adjacent to X as well as to Y), and $Z\notin S_{XY}$, place arrowheads pointing to Z, *i.e.*, X→Z←Y.

3. In the partially-oriented graph from step 2, orient as many edges as possible following two requirements:

(a) Any alternative orientation will not yield a new *V* structure (*i.e.*, X→Z←Y).

(b) Any alternative orientation will not yield a directed cycle.

In summary, we find all the pairs of variables that have a dependent relationship to reconstruct the basic structure of the underlying causal network in step 1. Then we find all the *V* structures in the network in step 2 and prevent the creation of new V structures or directed cycles in step 3.

### Marker effect decomposition

Estimated direct, indirect, and overall SNP effects from SEM-BayesCΠ incorporating the IC1 causal structure for the trait flowering time at Arkansas (FTA) are shown in Figure 5. The SNP “id1024159” has direct effect 0.005 on trait FTA, while its indirect effect transmitted through PH is -0.039. Thus, the overall effect of SNP “id1024159” is mainly determined by the indirect effect.

## References

[bib1] Anderson J. C., and Gerbing D. W., 1988 Structural equation modeling in practice: A review and recommended two-step approach. Psychol. Bull. 103: 411–423. 10.1037/0033-2909.103.3.411

[bib2] Begum F., Sharker M. H., Sherman S. L., Tseng G. C., and Feingold E., 2016 Regionally Smoothed Meta-Analysis Methods for GWAS Datasets. Genet. Epidemiol. 40: 154–160. 10.1002/gepi.21949 26707090 PMC4724289

[bib3] Cantor R. M., Lange K., and Sinsheimer J. S., 2010 Prioritizing GWAS Results: A Review of Statistical Methods and Recommendations for Their Application. Am. J. Hum. Genet. 86: 6–22. 10.1016/j.ajhg.2009.11.017 20074509 PMC2801749

[bib4] Chen C., Steibel J. P., and Tempelman R. J., 2017 Genome-Wide Association Analyses Based on Broadly Different Specifications for Prior Distributions, Genomic Windows, and Estimation Methods. Genetics 206: 1791–1806. 10.1534/genetics.117.202259 28637709 PMC5560788

[bib5] Cheng H., Garrick D. J., and Fernando R. L., 2018 a JWAS: Julia implementation of whole-genome analysis software. Proceedings of the World Congress on Genetics Applied to Livestock Production **11**: 859.

[bib6] Cheng H., Kizilkaya K., Zeng J., Garrick D., and Fernando R., 2018 b Genomic Prediction from Multiple-Trait Bayesian Regression Methods Using Mixture Priors. Genetics 209, 89–103. 10.1534/genetics.118.300650 29514861 PMC5937171

[bib7] Chicharro D., and Panzeri S., 2014 Algorithms of causal inference for the analysis of effective connectivity among brain regions. Front. Neuroinform. 8: 64 10.3389/fninf.2014.00064 25071541 PMC4078745

[bib8] Fernando R., Toosi A., Wolc A., Garrick D., and Dekkers J., 2017 Application of Whole-Genome Prediction Methods for Genome-Wide Association Studies: A Bayesian Approach. J. Agric. Biol. Environ. Stat. 22: 172–193. 10.1007/s13253-017-0277-6

[bib9] Fernando R. L., and Garrick D., 2013 Bayesian Methods Applied to GWAS, pp. 237–274 in Genome-Wide Association Studies and Genomic Prediction, Humana Press, Totowa, NJ 10.1007/978-1-62703-447-0_10 23756894

[bib10] Gianola D., 2013 Priors in Whole-Genome Regression: The Bayesian Alphabet Returns. Genetics 194: 573–596. 10.1534/genetics.113.151753 23636739 PMC3697965

[bib11] Gianola D., and Sorensen D., 2004 Quantitative Genetic Models for Describing Simultaneous and Recursive Relationships Between Phenotypes. Genetics 167: 1407–1424. 10.1534/genetics.103.025734 15280252 PMC1470962

[bib12] Hackinger S., and Zeggini E., 2017 Statistical methods to detect pleiotropy in human complex traits. Open Biol. 7: 170125 10.1098/rsob.170125 29093210 PMC5717338

[bib13] Korte A., Vilhjálmsson B. J., Segura V., Platt A., Long Q., 2012 A mixed-model approach for genome-wide association studies of correlated traits in structured populations. Nat. Genet. 44: 1066–1071. 10.1038/ng.2376 22902788 PMC3432668

[bib14] Liu H.-J., and Yan J., 2018 Crop genome-wide association study: a harvest of biological relevance. Plant J. 97: 8–18. 10.1111/tpj.14139 30368955

[bib15] Lloyd-Jones L. R., Robinson M. R., Moser G., Zeng J., Beleza S., 2017 Inference on the Genetic Basis of Eye and Skin Color in an Admixed Population via Bayesian Linear Mixed Models. Genetics 206: 1113–1126. 10.1534/genetics.116.193383 28381588 PMC5499166

[bib16] Ma H., Bandos A. I., Rockette H. E., and Gur D., 2013 On use of partial area under the ROC curve for evaluation of diagnostic performance. Stat. Med. 32: 3449–3458. 10.1002/sim.5777 23508757 PMC3744586

[bib17] McCarthy M. I., Abecasis G. R., Cardon L. R., Goldstein D. B., Little J., 2008 Genome-wide association studies for complex traits: consensus, uncertainty and challenges. Nat. Rev. Genet. 9: 356–369. 10.1038/nrg2344 18398418

[bib18] Mi X., Eskridge K., Wang D., Stephen Baenziger P., 2010 Bayesian mixture structural equation modelling in multiple-trait QTL mapping. Genet. Res. 92: 239–250. 10.1017/S0016672310000236 20667167

[bib19] Momen M., Campbell M. T., Walia H., and Morota G., 2019 Utilizing trait networks and structural equation models as tools to interpret multi-trait genome-wide association studies. Plant Methods 15: 107 10.1186/s13007-019-0493-x 31548847 PMC6749677

[bib20] Momen M., Mehrgardi A. A., Roudbar M. A., Kranis A., Pinto R. M., 2018 Including Phenotypic Causal Networks in Genome-Wide Association Studies Using Mixed Effects Structural Equation Models. Front. Genet. 9: 455 10.3389/fgene.2018.00455 30356716 PMC6189326

[bib21] Ozaki K., Ohnishi Y., Iida A., Sekine A., Yamada R., 2002 Functional SNPs in the lymphotoxin-alpha gene that are associated with susceptibility to myocardial infarction. Nat. Genet. 32: 650–654. 10.1038/ng1047 12426569

[bib22] O’Reilly P. F., Hoggart C. J., Pomyen Y., Calboli F. C. F., Elliott P., 2012 MultiPhen: Joint Model of Multiple Phenotypes Can Increase Discovery in GWAS. PLoS One 7: e34861 10.1371/journal.pone.0034861 22567092 PMC3342314

[bib23] Pearl J., 2009 Causal inference in statistics: An overview. Stat. Surv. 3: 96–146. 10.1214/09-SS057

[bib24] Scutari M., 2014 Bayesian Network Constraint-Based Structure Learning Algorithms: Parallel and Optimised Implementations in the bnlearn R Package.

[bib25] Sharma A., Lee J. S., Dang C. G., Sudrajad P., Kim H. C., 2015 Stories and Challenges of Genome Wide Association Studies in Livestock - A Review. Asian-Australas. J. Anim. Sci. 28: 1371–1379. 10.5713/ajas.14.0715 26194229 PMC4554843

[bib26] Sing T., Sander O., Beerenwinkel N., and Lengauer T., 2005 ROCR: visualizing classifier performance in R. Bioinformatics 21: 3940–3941. 10.1093/bioinformatics/bti623 16096348

[bib27] Sodini S. M., Kemper K. E., Wray N. R., and Trzaskowski M., 2018 Comparison of Genotypic and Phenotypic Correlations: Cheverud’s Conjecture in Humans. Genetics 209: 941–948. 29739817 10.1534/genetics.117.300630 PMC6028255

[bib28] Song X.-Y., and Lee S.-Y., 2012 A tutorial on the Bayesian approach for analyzing structural equation models. J. Math. Psychol. 56: 135–148. 10.1016/j.jmp.2012.02.001

[bib29] Valente B. D., Rosa G. J. M., Campos G., Gianola D., and Silva M. A., 2010 Searching for Recursive Causal Structures in Multivariate Quantitative Genetics Mixed Models. Genetics 185: 633–644. 10.1534/genetics.109.112979 20351220 PMC2881143

[bib30] VanRaden P., 2008 Efficient Methods to Compute Genomic Predictions. J. Dairy Sci. 91: 4414–4423. 10.3168/jds.2007-0980 18946147

[bib31] Visscher P. M., Wray N. R., Zhang Q., Sklar P., McCarthy M. I., 2017 10 Years of GWAS Discovery: Biology, Function, and Translation. Am. J. Hum. Genet. 101: 5–22. 10.1016/j.ajhg.2017.06.005 28686856 PMC5501872

[bib32] Wright S., 1921 Correlation and Causation. J. Agric. Res. 557–585.

[bib33] Wright S., 1934 The Method of Path Coefficients. Ann. Math. Stat. 5: 161–215. 10.1214/aoms/1177732676

[bib34] Wu X., Heringstad B., and Gianola D., 2010 Bayesian structural equation models for inferring relationships between phenotypes: a review of methodology, identifiability, and applications. J. Anim. Breed. Genet. 127: 3–15. 10.1111/j.1439-0388.2009.00835.x 20074182

[bib35] Zhao K., Tung C.-W., Eizenga G. C., Wright M. H., Ali M. L., 2011 Genome-wide association mapping reveals a rich genetic architecture of complex traits in Oryza sativa. Nat. Commun. 2: 467 10.1038/ncomms1467 21915109 PMC3195253

